# Subjective expansion of extended time-spans in experienced meditators

**DOI:** 10.3389/fpsyg.2014.01586

**Published:** 2015-01-14

**Authors:** Marc Wittmann, Simone Otten, Eva Schötz, Anna Sarikaya, Hanna Lehnen, Han-Gue Jo, Niko Kohls, Stefan Schmidt, Karin Meissner

**Affiliations:** ^1^Institute for Frontier Areas in Psychology and Mental HealthFreiburg, Germany; ^2^Institute of Medical Psychology, Ludwig-Maximilian University MunichMunich, Germany; ^3^Department of Psychosomatic Medicine, University Medical Center FreiburgFreiburg, Germany; ^4^Institute for Transcultural Health Studies, European University ViadrinaFrankfurt (Oder), Germany; ^5^Division Integrative Health Promotion, University of Applied SciencesCoburg, Germany

**Keywords:** mindfulness meditation, time perception, passage of time, time perspective, impulsiveness

## Abstract

Experienced meditators typically report that they experience time slowing down in meditation practice as well as in everyday life. Conceptually this phenomenon may be understood through functional states of mindfulness, i.e., by attention regulation, body awareness, emotion regulation, and enhanced memory. However, hardly any systematic empirical work exists regarding the experience of time in meditators. In the current cross-sectional study, we investigated whether 42 experienced mindfulness meditation practitioners (with on average 10 years of experience) showed differences in the experience of time as compared to 42 controls without any meditation experience matched for age, sex, and education. The perception of time was assessed with a battery of psychophysical tasks assessing the accuracy of prospective time judgments in duration discrimination, duration reproduction, and time estimation in the milliseconds to minutes range as well with several psychometric instruments related to subjective time such as the Zimbardo Time Perspective Inventory, the Barratt Impulsivity Scale and the Freiburg Mindfulness Inventory. In addition, subjective time judgments on the current passage of time and retrospective time ranges were assessed. While subjective judgements of time were found to be significantly different between the two groups on several scales, no differences in duration estimates in the psychophysical tasks were detected. Regarding subjective time, mindfulness meditators experienced less time pressure, more time dilation, and a general slower passage of time. Moreover, they felt that the last week and the last month passed more slowly. Overall, although no intergroup differences in psychophysical tasks were detected, the reported findings demonstrate a close association between mindfulness meditation and the subjective feeling of the passage of time captured by psychometric instruments.

## INTRODUCTION

One aspect that is noticed by individuals during mindfulness-oriented meditation and thereafter in daily life is that they frequently experience subjective time slowing down considerably and the present moment feels expanded (Kabat-Zinn, 2005). Conceptually, the ability of expert mindfulness meditators to focus more strongly on momentary sensory experiences and to be increasingly aware of one’s own feelings and body states leads to a slowing down of time and an expansion of the experienced present moment (Wittmann and Schmidt, 2014). According to a recent conceptualization, self- and bodily processes lie at the core of human time perception, and intertwined affective and interoceptive states create our experience of time (Craig, 2009; Droit-Volet et al., 2013; Wittmann, 2013; Pollatos et al., 2014a,b). These functional aspects are also affected by states of mindfulness meditation, namely through self-awareness and self-regulation encompassing bodily awareness and emotion regulation (Hölzel et al., 2011; Vago and Silbersweig, 2012; Kerr et al., 2013; Esch, 2014). An increased attentional capacity to focus on the present moment in mindfulness meditators (Jha et al., 2007; Zeidan et al., 2010; Moore et al., 2012) thereafter might influence time judgments of momentarily experienced temporal intervals (prospective time estimation). In addition, retrospective estimation of duration might be affected through an increased awareness of events at the present moment which in turn leads to enhanced build-up of episodic memory load (Wittmann and Schmidt, 2014). For the distinction of cognitive models concerning prospective and retrospective duration judgment, see the overview by Zakay and Block (1997). In essence, in this family of models, prospective time is explained according to a pacemaker–accumulator where a pacemaker produces a series of ‘pulses,’ analogous to the ticks of a clock. The number of pulses recorded during an interval represents experienced duration. However, only when attention is actually directed to time an attentional gate opens and allows the pulses to accumulate. The more attention is paid to the passage of time the longer duration is experienced (Droit-Volet and Meck, 2007). In retrospective time perception duration is reconstructed from memory. That is, the more contextual changes are perceived during a certain time span, which are stored in memory and later retrieved, the longer the duration is subjectively experienced in retrospect (Block and Zakay, 1997; Avni-Babad and Ritov, 2003). However useful these cognitive models of subjective time may be, regarding neural underpinnings of time perception no consensus exists, many different functional and neurophysiological models exist (Wittmann and van Wassenhove, 2009; Hancock and Block, 2012; Merchant et al., 2013; Wittmann, 2013).

The study of individuals in altered states of consciousness or with specially trained capacities such as in expert meditators offers a way to explore the psychological and neural mechanisms underlying both time perception and consciousness. In altered states of consciousness such as in meditative states, under the influence of drugs or in many psychiatric and neurological conditions, the sense of the self is modulated together with the time sense (for an earlier overview, see Block, 1979). In vivid day dreaming or in hypnoses where individuals imagine scenes and are not focused on themselves duration is typically underestimated (Naish, 2007). On the other hand, individuals during mindfulness meditation as well as in everyday life state that subjective time slows down and felt duration expands (Kabat-Zinn, 2005). Only few studies to date exist however which have attempted to empirically disclose the relationship between mindfulness and the perception of time. In one study assessing the duration of the present moment as operationalized with the perceptual switching of the Necker cube, a visual ambiguous figure, 38 practitioners of mindfulness showed longer duration of subjective ‘nowness’ as compared to 38 control subjects with no meditation experience in the instruction condition to hold a given percept as long as possible (Sauer et al., 2012). That is, the proficiency of mindfulness was found to be associated with an increased ability to stabilize an ambiguous percept in a bi-stable image paradigm. It was concluded that the subjective “now” can be longer for meditators than for non-meditators, and individual levels of mindfulness may convey this effect. This finding complements an earlier study with Buddhist monks who during their lifetime have extensive meditation practice on a daily basis and were able to voluntarily prolong the mental perception of one out of two alternations in the binocular rivalry paradigm (Carter et al., 2005). In a study with a population of unselected students (Wittmann et al., 2014), scoring higher on the Freiburg Mindfulness Inventory (FMI; Walach et al., 2006) and the Comprehensive Inventory of Mindfulness Experience (CHIME; Bergomi et al., 2013) was found to be related to a better emotional handling of the past and a more pronounced future perspective. Self-attributed mindfulness levels were associated with a more accurate timing ability in the milliseconds and multiple seconds range. These findings suggest a close association between dispositional or trait mindfulness and the perception of time.

Only a few studies exist so far which investigated how time perception in the seconds-to-minutes range is altered before and after meditation experience, thus probing for induced states of mindfulness. In one study, subjects with no experience in meditation took part in a 1-week mindfulness meditation program with one 30-min meditation session per day (Sucala and David, 2013). At the end of the week, the meditation group relatively over-estimated the duration of presented video-clips which lasted 5-min as compared to a group that had not participated in the program. However, no direct pre- vs. post meditation task was applied and no group with a control program was tested. A pre- post design and the comparison with a control task group was undertaken in a different study, in which subjects with no experience in meditation were exposed to a pre-recorded meditation session of 10 min duration (Kramer et al., 2013). Using the temporal bisection task in the time range around 1 s, it was shown that the meditation group relatively overestimated the duration of stimuli post- vs. pre- meditation as well as compared to the control group, which did not show a difference before vs. after listening to a story for 10 min. In a recent study using the temporal bisection task with durations between 4 and 8 s, mindfulness meditation experience increased the sensitivity to time and lengthened subjective duration. This temporal improvement was primarily observed in more experienced meditators as compared to meditation-naïve individuals (Droit-Volet et al., 2015). Another study has correlated time perception performance with brain activation correlates (EEG) and found a decrease of gamma power in midline structures together with longer duration productions (Berkovich-Ohana et al., 2012). This duration production task in the seconds’ range actually showed to be sensitive for differences between meditation techniques when assessing pre- post meditation changes in timing performance (Berkovich-Ohana et al., 2011). Individual highly trained meditators after years of practice can eventually reach altered states of consciousness during meditation where the experiences of time and space together with a sense of self are lost (Berkovich-Ohana et al., 2013). However, in our study we concentrate on possible differences in subjective time between meditators and non-meditators in every-day experience and behavior.

So far only a narrow selection of time perception tests has been employed in cross-sectional or longitudinal studies probing for differences in trait-mindfulness or after practicing meditation. Moreover, only prospective time judgments in the seconds-to-minutes range have been assessed. Here, we present results from a cross-sectional study, where we systematically investigated whether individuals with extensive mindfulness meditation training are more precise and accurate in psychophysical time perception tasks assessing different modalities and different time ranges in a series of prospective time judgment tasks. We employed those temporal processing tasks which not only have proven to reveal inter-individual differences in various subject groups (Wittmann et al., 2011; Pütz et al., 2012), but in a recent study also have shown to be sensitive to variations in self-rated mindfulness (Wittmann et al., 2014). Moreover, we wanted to use a selection of classic tasks (duration discrimination, duration reproduction, duration production, time estimation) covering a range of durations from the milliseconds to multiple seconds. Subjective time was furthermore assessed with subjective scales on the felt passage of time and prospective and retrospective judgments of longer periods of time, i.e., the day, the week, years. Moreover, we employed inventories assessing the time perspectives of the past, present, and future. In order to assess an extensive profile of psychological factors, which has shown to be related to subjective time, we also measured momentary subjective well-being, general physical activity, and personality indices such as subjective ratings of mindfulness, impulsiveness, as well as objective indices of attentional capacities (Wittmann et al., 2014). The latter capacity is included in this study since attention strongly modulates time perception (Droit-Volet and Meck, 2007) and attention is affected by mindfulness meditation practice (Jha et al., 2007; Wittmann and Schmidt, 2014).

## MATERIALS AND METHODS

### DESIGN

We performed a cross-sectional study with one measurement point comparing experienced meditators with matched controls. The study was conducted in two laboratories, one in Freiburg, Germany, and one in Munich, Germany, in a multi-center approach in order to raise the validity of the results. The study was approved by the Ethics committees of the two Universities.

### PARTICIPANTS

42 experienced mindfulness meditation practitioners (at least 3 years of continuous practice; at least 2 h of practice over the last 8 weeks) and 42 age-, sex and education case matched controls, without any meditation experience, were investigated. Subjects were recruited by advertisement in meditation centers, on online platforms of the universities, and by word of mouth. Meditators were included when practicing regularly a form of meditation which had a dominant orientation toward awareness of the present moment. Therefore we included individuals if they participated in forms of mindfulness meditation, Vipassana meditation, or Soto Zen. Age was restricted to the range between 21 and 50 years in order to constrain age-related effects in the psychophysical tasks. Control subjects were supposed to have no experience with any form of meditation including Yoga or Tai-Chi. The matching criteria were sex, age (±5 years) and education (±1 level of 5). Participants who had to be fluent in German were additionally required to report good health and no known medical, psychological, neurological or psychiatric problems as assessed with a detailed screening form. Participants signed an informed consent and received a moderate financial compensation (€20) for participation which lasted ~2 h.

### INSTRUMENTS

Two inventories were employed to control for potential factors that could influence and explain performance and subjective ratings between groups: the German version of the international physical activity questionnaire (IPAQ; Ainsworth et al., 2000) assesses the amount of physical activity over the last week; with the *Kurzfragebogen zur aktuellen Beanspruchung* (short questionnaire on current demands, KAB; Müller and Basler, 1993) the awareness of everyday demands (and resulting stress level) is assessed; this inventory thus can be used as a proxy measure for the momentary well-being of an individual. Both physical activity (Sysoeva et al., 2013) and emotional well-being (Lamotte et al., 2014) have shown to influence time judgments.

#### Freiburg Mindfulness Inventory

The 14-item version of the FMI (Walach et al., 2006; Kohls et al., 2009) measures mindfulness on the basis of a two-dimensional structure with the factor ‘presence’ as ability to attend to the present moment (“I am open to the present moment”) and the factor ‘acceptance’ as non-judgmental attitude (“I am able to smile when I notice how I sometimes make life difficult”) utilizing a 4-point item scale format.

#### The Barratt Impulsiveness Scale (BIS-11)

The German version (Preuss et al., 2008) of the BIS-11 (Barratt et al., 1999) consists of 30 4-point items ranging from 1 (rarely) to 4 (almost always). The resulting subscales are planning impulsivity (“I plan tasks carefully”), motor impulsivity (“I do things without thinking”), and attention/cognition impulsivity (“I concentrate easily”).

#### Zimbardo Time Perspective Inventory (ZTPI)

The German version (Brandler and Rammsayer, 2002) of the ZTPI (Zimbardo and Boyd, 1999) contains 56 5-point items ranging from 1 (very untrue) to 5 (very true). Five subscales represent the individual focus on the time perspectives: past-negative (“I think about the bad things that have happened to me in the past”), present-hedonistic (“I take risks to put excitement in my life”), future (“I am able to resist temptations when I know that there is work to be done”), past-positive (“Happy memories of good times spring readily to mind”), and present-fatalistic (“My life path is controlled by forces I cannot influence”).

#### Subjective time questionnaire

The first part of the subjective time questionnaire (Wittmann and Lehnhoff, 2005) consists of questions concerning the subjective experience of the passage of time in the present, past, and future and is assessed with visual analog scales with the two anchor points: “very slowly” and “very fast.” The first question asks: “How fast does time usually pass for you?” and is potentially related to an intermediate time range associated with the course of hours and the present day. The retrospective perspective of longer time intervals is assessed with four questions asking how fast last week, last month, last year, and the past 10 years have passed. Four questions assess the outlook concerning future time intervals of next week, next month, next year, and the next 10 years. All of the visual analog scales had a length of 100 mm and answers were read out at the point (in millimeters) subjects marked on the horizontal line. The second part of the questionnaire consists of statements concerning subjective judgements of time with assigned values of 0 = strong rejection to 4 = strong approval referring to the feeling of time pressure (five statements, e.g., “I often think that time is running out”) or to the feeling of time expansion (five statements, e.g., “My time is not filled”). A mean value was calculated for the five statements on ‘time pressure’ and ‘time expansion.’

#### Time estimation and time production

In three different computerized number reading tasks compiled by a PowerPoint presentation, subjects had to estimate the duration of the tasks (prospective verbal time estimation), that is, reading aloud numbers presented on the screen with jittered pause intervals of 4, 6, or 8 s. In the two estimation tasks subjects had to estimate the duration of two number reading sessions of 40 and 80 s. In the production task subjects were requested to press a button when they felt that 1 min had passed. Essentially, number reading in the three tasks was designed to prevent subjects from counting. The actual task was to estimate and produce the durations as accurately as possible. Each of the tasks was presented once.

#### Auditory duration discrimination

In the employed computer task (Pütz et al., 2012) subjects had to indicate which one out of two presented auditory stimuli (white noise) was longer, the first or the second. In the sequence of trials the standard duration was always 1000 ms, the comparison stimulus was always longer and separated by a pause interval of 500 ms. Standard and comparison stimuli were presented in random order. The longer stimulus varied according to the adaptive maximum-likelihood based algorithm YAAP (Treutwein, 1997), which determines the next stimulus presentation according to the subject’s responses. According to this procedure, stimuli in each trial are set at the current best estimate of the threshold. This tracking procedure estimates a threshold – representing duration discrimination accuracy – corresponding to 75% correct discrimination based on a logistic psychometric function. According to this procedure the number of trials varies between subjects and can be taken as measure of precision over the course of given responses.

#### Visual and auditory duration reproduction – long

In two separate computer tasks running on Psychtoolbox for Matlab subjects had to reproduce the duration of (1) a visual and (2) an auditory stimulus with intervals of 8, 14, and 20 s duration (six trials per interval, respectively, presented in randomized order). In each trial, a green square (a sinus tone of 440 Hz) is first presented for one of the three durations. After the pause of either 4.5, 5, or 5.5 s duration a second yellow square (a sinus tone of 500 Hz) appeared. It had to be stopped by pressing the space bar when participants felt that this second stimulus had reached the duration of the first. Participants were requested not to use mental strategies such as inner counting but to rely on their subjective feeling of elapsed time. To further prevent mental counting, participants were additionally given a secondary working-memory task. Before each trial four numbers were presented. At the end of each trial one number appeared and the subjects responded whether the number was one of the four previously presented by pressing the left or right arrow button, respectively for ‘yes’ and ‘no.’ The accuracy and precision of duration reproduction can be calculated from the temporal reproduction performance. In a subset of subjects we additionally recorded psychophysiological parameters, results of which will be presented elsewhere.

#### Visual duration reproduction – short

Similar to the two versions with longer intervals, a visual (but not auditory) duration reproduction task with stimulus duration of 600, 800, 1000, 1200, and 1400 ms (with eight trials per interval presented in randomized order) was employed with a green and yellow square marking the two intervals and with pause intervals of 1.5, 2.75, 2, 2.25, or 2.5 s in between. No secondary working-memory task was presented for these short stimuli. Subjects were requested to stop the second yellow square by pressing the space bar when subjects felt that it had reached the duration of the first green square.

#### The attention network test (ANT)

The ANT (Fan et al., 2002) assesses the processing efficiency of the three attention networks of (1) alerting, (2) orienting, and (3) executive attention. They are quantified by means of computerized reaction time measures for differently cued and un-cued stimulus conditions with or without presented flankers. In essence, the ANT is a combination of a cued reaction time task and a flanker task, where cues can facilitate reaction time and flankers can either facilitate or impede reaction time. Participants have to respond to either the left or right arrow key on the computer keyboard depending on the stimulus configuration, the target stimulus being an arrow pointing to the left or right and flankers being either congruent (facilitating) or incongruent (impeding). An overall reaction time score and an index of accuracy are also calculated.

### STATISTICAL ANALYSES

*t*-tests for group differences are employed and one-factorial ANOVAs for group differences with the two factors of ‘group’ and the different ‘time intervals’ in the duration reproduction tasks. For multiple comparisons alpha level adjustments according to the false discovery rate method (FDR; Benjamini and Hochberg, 1995) was applied for each measurement and inventory with initial significance levels set to *p* < 0.05. When statistically significant group differences are found, we calculated the effect size using Cohen’s *d*.

## RESULTS

The 42 meditators (17 men) with a mean age of 40.2 years (SD = 7.6; range = 24–50) had a meditation experience of on average 10.4 years (SD = 6.9; range: 3–32 years) and had practiced on average 7.4 h per week (SD = 5.0; range: 2–21 h) over the last 8 weeks. See **Table 1** for all the following performance indices of the two groups and related statistics. No group differences were detected in the amount of physical activity over the last week as assessed with the International Physical Activity Questionnaire [*t*(82) = -0.128; *p* = 0.205] and in the awareness of everyday demands resulting in varying stress levels assessed with the KAB [*t*(82) = -0.028; *p* = 0.978]. Meditators had significantly higher values of mindfulness in the FMI in both subscales of ‘presence’ [*t*(82) = -5.624; *p* = 0.0001; Cohen’s *d* = -1.23] and ‘acceptance’ [*t*(82) = -6.614; *p* = 0.0001; Cohen’s *d* = -1.44]. However, they did not differ in any of the five variables of the Attention Network Test (ANT; all *p* > 0.1). Surprisingly, meditators reported more impulsiveness in the Barratt Impulsiveness Scale on one subscale [‘BIS motor impulsivity’: *t*(82) = -2.960; *p* = 0.004; significant after FDR adjustment; Cohen’s *d* = 0.65; see also BIS sum: *t*(82) = -2.266; *p* = 0.026; n.s.; ‘BIS non-planning impulsivity’: *t*(82) = -2.442; *p* = 0.017; n.s.].

**Table 1:** Mean group differences between meditators and control subjects in self-rating scales.

	Mean (SD)			
Variable	Mindfulness meditators	Control subjects	*T* (df = 82)	*p*
Physical activity questionnaire (IPAQ)	3678 (*2635*)	3015 (*2086*)	-1.277	0.205
Awareness of everyday demands (KAB)	13.9 (*3.6*)	13.9 (*4.1*)	-0.028	0.978
**Freiburg Mindfulness Inventory (FMI)**
Presence	**19.4 (*1.9*)**	**16.5 (*2.8*)**	**-5.624**	0.0001*
Acceptance	**24.9 (*3.1*)**	**20.2 (*3.5*)**	-**6.614**	0.0001*
**Attention Network Test (ANT)**
Alerting	33.1 (*18.9*)	34.5 (*23.5*)	0.286	0.775
Orienting	54.2 (*24.1*)	51.1 (*25.9*)	-0.566	0.573
Executive attention	116.3 (*28.7*)	125.0 (*38.1*)	1.192	0.237
Mean Reaction time [ms]	579 (*67*)	601 (*59*)	1.628	0.107
Mean accuracy [%]	96.6 (*7.1*)	96.8 (*7.2*)	0.137	0.891
**Barratt Impulsiveness Scale (BIS)**
Non-planning	26.0 (*4.4*)	23.6 (*4.6*)	-2.442	0.017
Motor	**24.9** (*4.5*)	**21.9** (*4.6*)	**-2.960**	0.004*
Cognitive	**14.6** (*3.1*)	**17.1** (*10.4*)	1.502	0.137
BIS Sum	65.3 (*7.9*)	61.2 (*8.8*)	-2.266	0.026
**Zimbardo Time Perspective Inventory (ZTPI)**
Past negative	**21.6 (*4.8*)**	**24.8 *(5.7)***	**2.792**	0.007*
Present hedonistic	50.3 (*5.7*)	48.7 (*7.4*)	-1.107	0.272
Future	41.4 (*5.7*)	43.9 (*6.8*)	1.848	0.068
Past positive	29.4 (*4.3*)	31.3 (*4.5*)	1.928	0.057
Present fatalistic	23.9 (*4.6*)	21.7 (*4.2*)	-2.308	0.024
**Subjective time questionnaire [0 ... 100]**
“How fast does time usually pass for you?”	** 61 *(16)***	** 75 (*14*)**	**4.117**	0.0001*
**How fast …**
… last week	**64 (*18*)**	**78 *(18)***	**3.599**	0.001*
… last month	**62 *(19)***	**77 *(16)***	**3.768**	0.0001*
… last year	65 (*19*)	72 (*21*)	1.665	0.1
… last 10 years	59 (*21*)	68 (*21*)	1.871	0.065
… next week	**58 (*20*)**	**73 (*21*)**	**3.492**	0.001*
… next month	**60 *(18)***	**69 *(18)***	**2.338**	0.022*
… next year	55 (*19*)	**67 *(19)***	**2.821**	0.006*
… next 10 years	57 (*22*)	63 (*22*)	1.254	0.213
Time pressure [0 … 4]	**2.00 (*0.8*)**	**2.69 (*0.7*)**	**4.224**	0.001*
Time expansion [0 … 4]	**0.79 (*0.6*)**	**0.46 (*0.4*)**	**-2.843**	0.006*

*Significant differences with initial alpha level of 0.05 adjusted with the false discovery rate (FDR) for multiple tests marked in bold.

In the Zimbardo Time Perspective Inventory (ZTPI) control subjects showed significantly higher values in the past negative perspective compared to meditators [*t*(82) = 2.792; *p* = 0.007; Cohen’s *d* = -0.61]; no other variables of the ZTPI revealed significant group differences. In the subjective time questionnaire assessing the passage of time, several parameters showed significant group differences in the way that time was relatively slowed down in meditators and temporal intervals were relatively expanded: compared to controls, meditators felt less time pressure [*t*(82) = 4.224; *p* = 0.0001; Cohen’s *d* = -0.92], more time dilation [*t*(82) = 2.843; *p* = 0.006; Cohen’s *d* = 0.62], and a generally slower passage of time [*t*(82) = 4.117; *p* = 0.0001; Cohen’s *d* = -0.9]; moreover, they sensed that the past week [*t*(82) = 3.599; *p* = 0.001; Cohen’s *d* = -0.79] and the past month [*t*(82) = 3.768; *p* = 0.0001; Cohen’s *d* = -0.82] were subjectively longer; but not the last year and the last 10 years. Judging prospectively, meditators thought that the next week [*t*(82) = 3.492; *p* = 0.001; Cohen’s *d* = -0.76], the next month *t*(82) = 2.338; *p* = 0.022; Cohen’s *d* = 0.51], the next year [*t*(82) = 2.822; *p* = 0.006; Cohen’s *d* = -0.62; all significant after FDR adjustment], but not the next 10 years, would be passing by more slowly.

In the psychophysical time perception tasks spanning the range from milliseconds (auditory duration discrimination, visual duration reproduction) to several seconds (visual and auditory duration reproduction), and the minutes range (time estimation and time production), no group differences for ‘accuracy’ or ‘precision’ of performance between the mindfulness meditators and the matched controls were detected (see **Table 2** for performance indices), nor were any interactions of group × intervals in the temporal reproduction tasks significant (*t*-tests and ANOVAs all *p* > 0.1).

**Table 2:** Mean group differences between meditators and control subjects in psychophysical tasks.

	Mean (SD)			
Variable	Mindfulness meditators	Control subjects	*TF* (df = 82)	*p*
**Psychophysical timing tasks**
Auditory duration discrimination threshold [ms]	160 (*65*)	165 (*82*)	-0.360	0.720
Visual duration reproduction – short [ms]				
600 ms	622 (*124*)	616 (*124*)	F = 0.262	0.816
800 ms	834 (*155*)	806 (*131*)		
1000 ms	1017 (*158*)	984 (*131*)		
1200 ms	1153 (*189*)	1124 (*167*)		
1400 ms	1307 (*216*)	1286 (*202*)		
**Visual duration reproduction – long [s]**
8 s	7.990 *(2.048)*	7.771 (*1.654*)	F = 0.593	0.554
14 s	12.369 *(2.636)*	12.575 (*2.311*)		
20 s	16.617 (*3.146*)	16.690 (*2.541*)		
**Auditory duration reproduction – long [s]**
8 s	7.442 *(1.435)*	7.262 (*1.670*)	F = 0.203	0.719
14 s	11.786 *(1.827)*	11.345 *(1.826)*		
20 s	15.715 *(3.268)*	15.585 (*3.174*)		
Duration estimation 40 s	66 (*32*)	67 (*32*)	0.120	0.905
Duration estimation 80 s	108 (*42*)	106 (*41*)	-0.217	0.829
Duration production 60 s	54 (*21*)	61 (*21*)	1.642	0.104

## DISCUSSION

We compared performance of 42 experienced mindfulness meditators and 42 age-, sex-, and education matched controls. On average, the included meditators were comparatively highly experienced in mindfulness meditation since they had practiced on average for 10 years and had practiced regularly for an average of 7 h per week over the last 8 weeks. With respect to time perception indices, the focus of our investigation, the results are clear-cut: regarding subjective time, mindfulness meditators experienced less time pressure, more time dilation, and a slower passage of time. Moreover, in retrospective judgments, they felt that the last week and the last month had passed more slowly. However, and importantly, regarding psychophysical tasks in the range of milliseconds-to-seconds and to minutes, there were no group differences regarding accuracy and precision of timing performance. We will discuss these results below.

Results relating to group differences of the FMI, ZTPI, and BIS will be considered first as they define the context and understanding of the above mentioned results in subjective time. Not surprisingly, the group of meditators reported to be more mindful with respect to both factors of the FMI of ‘presence’ and ‘acceptance.’ In the ZTPI control subjects loaded higher on the ‘past negative’ subscale, no other time perspective revealed significant differences. As has been shown before, a non-judgmental attitude – which is one focus of mindfulness meditation practice – is related to more emotional control, which again is associated with more positive attitudes concerning past events – as measured with the ZTPI (Wittmann et al., 2014). Surprisingly, the meditation group exhibited significantly higher values in the subscale of ‘motor impulsiveness.’ Typically, ‘mindfulness’ and ‘impulsiveness’ are negatively correlated. In studies using different self-rating and behavioral assessment techniques, higher impulsivity/less self-control in individuals is related to lower scores of mindfulness (Peters et al., 2011; Bowlin and Baer, 2012; Lee and Chao, 2012; Wittmann et al., 2014). This is conceptually not surprising since ‘impulsiveness’ can be seen as lying on one end of a conceptual continuum with ‘self-control’ and ‘mindfulness’ forming the other end (Sauer et al., 2011; Wittmann et al., 2014). There are at least two possibilities as to why the experienced meditators in our study showed higher impulsivity scores. For one, it is possible that in the selection process of our study we not only found individuals who due to their meditation experience are more self-controlled. We may also have included individuals who as a result of experiencing more impulsiveness became regular meditators. That is, they would meditate as a form of psychological “self-therapy.” Since more impulsive individuals are less accurate in psychophysical time perception tasks than more self-controlled subjects (Wittmann and Paulus, 2008; Wittmann et al., 2011), the fact that our meditators on average were more impulsive may explain why we did not find group differences in the employed psychophysical tasks, which covered a broad range of methods and time scales.

However, an unintended side-effect lies in the possibility that meditators interpreted certain items in the BIS in a conceptually different way. That is, they would score higher on certain items of the motor impulsivity subscale due to being more mindful but not being more impulsive. We came to this conclusion by inspection of individual items of the BIS. In order to statistically test this idea we ran separate *t*-tests for each individual item in the subscale of motor impulsivity. The BIS items 4 [*t*(82) = -3.009; *p* = 0.003], 17 [*t*(82) = -3.008; *p* = 0.003], and 19 [*t*(82) = -4.438; *p* = 0.0001] were significantly higher scored by the meditation group than by the control group. As we will see, there might even be a translation issue at stake here. The item 4 (“I am happy-go-lucky”; in German: “Ich bin unbekümmert”), item 17 (“I act on impulse”; “Ich bin spontan”), and item 19 (“I act on the spur of the moment”; “Ich handle aus dem Moment heraus”) in the German translations may load highly in meditators because they imply that someone lives within the present moment. This conceptual interpretation is strengthened by the German translation of “I act on impulse,” which could have been “Ich handle impulsiv,” but was translated into “Ich bin spontan” which means “I am spontaneous.” Thus we conclude that the BIS items are ambiguous in the sense that a more mindful individual might also be coded as being more impulsive although he or she is not. The conceptual background of meditators thereafter might have led to higher scores in the BIS that are not necessarily related to actual higher impulsiveness.

The main results of our study pertaining to time perception are twofold: (1) long-term meditation practice can slow down the subjective passage of time as well as prolong the retrospective sense of past intervals; (2) psychophysical performance of time perception in the milliseconds-to-seconds and minutes range did not differ between meditators and controls. These two result clusters are related to two fundamental perspectives in time perception, namely prospective and retrospective estimates of duration (Zakay and Block, 1997; Grondin, 2010). In prospective timing tasks participants have to judge the duration of an interval in the seconds-to-minutes range they are presently experiencing. Time perception is thereafter assessed as relation between subjective estimates of duration and objective clock time. We had expected to find better performance of meditators in the prospective psychophysical timing tasks for two reasons: (a) practice in mindfulness meditation has shown to enhance attentional capacities and working memory (Jha et al., 2007; Zeidan et al., 2010; Moore et al., 2012; Leonard et al., 2013; Malinowski, 2013), these mental faculties being strongly involved in time perception of shorter duration (Block and Zakay, 1997; Grondin, 2010); (b) the perception of time within the present moment is strongly embodied as it is related to the interoceptive system and to feeling states of the body (Craig, 2009; Droit-Volet et al., 2013; Wittmann, 2013), mindfulness meditation strengthening body awareness and increasing the accuracy in discriminating body sensations (Fox et al., 2012; Mirams et al., 2013).

Related to our discussion of finding higher values in self-rated impulsivity in the group of meditators and the potential reason of having included individuals who might use meditation as a form of psychological self-regulation, we may have tested a group of meditators that did not outperform the control subjects. For example, our meditating group did also not perform differently in the employed computerized attention task (ANT). This attention task has shown to be sensitive to individual performance progress directly after meditation exposure (Tang et al., 2007) or in an assessment following a mindfulness training program lasting several weeks but not directly after a meditation session (Jha et al., 2007). That is, improvements in performance have so far mainly been detected in meditation studies with pre- post task differences, for example in studies probing changes in time perception (Kramer et al., 2013; Droit-Volet et al., 2015). The lack of differences in the psychophysical tasks between the two groups is potentially related to large inter-individual differences in participants. Therefore, investigations on intra-individual differences before and after meditation practice (directly after meditation or following mindfulness training programs) will yield stronger effects than our cross-sectional approach.

However we found clear-cut group differences in the retrospective subjective experience of time. ‘Retrospective judgments’ mean that subjective duration is not presently experienced – as is the case in the prospective psychophysical task – but is reconstructed from long-term memory. That is, retrospective duration depends on the amount of experienced contextual changes stored in memory. The more experienced changes have been stored, the longer an interval is judged to have lasted (Block and Zakay, 1997; Flaherty et al., 2005; Wittmann and Lehnhoff, 2005; Bailey and Areni, 2006). In retrospective judgments of time, longer intervals can be judged such as the last week, month, year and the last 10 years. Related, the question of “how fast does time usually pass for you” and the anticipated intervals of the next week, month, year and the next 10 years are based on retrospective experiences of past time intervals (Yi et al., 2009). The explanation of why retrospective time intervals in experienced meditators are subjectively prolonged is the following: since individuals trained in mindfulness meditation are more strongly aware of sensory events, their retrospective judgment of past intervals will likely lead to the impression of longer duration and a slowing down of subjective time (Wittmann and Schmidt, 2014). Mindfulness meditation is the enhanced awareness of the present moment as an embodied self. But also in ever-day life a practitioner focuses on her/his experiences more strongly (Kabat-Zinn, 2005). The ability of mindful persons in an every-day context to focus more strongly on sensory experiences and to be more strongly aware of feelings and of body states – essentially being aware of what is happening right now – causes more experiences to be stored in episodic memory which in turn leads to a relative expansion of retrospective duration (Block and Zakay, 1997; Avni-Babad and Ritov, 2003; Flaherty et al., 2005). That is, an increased focus on the feeling self and on the perceived environment at the present moment generally slows down the subjective passage of time in individuals who practice mindfulness. This is assessed with the question of “How fast does time usually pass for you?” and the questions concerning ‘time pressure’ and ‘time dilation’ where meditators consistently reported a slower passage of time. But also in retrospect, as a consequence of an increased awareness in the ‘here and now,’ which leads to more contextual changes in memory, and related to the questions of how long the last week and the last month lasted, meditators report a slower passage of time. Naturally, this explanation is only theoretical since in our study we did not investigate whether meditators actually had enriched episodic long-term memory or better retrieval mechanisms. It has though been shown that mindfulness meditation practice actually improves retrieval of positive emotions from memory (Roberts-Wolfe et al., 2012). Moreover, one could argue that having an increased focus in the present moment should also lead to better timing abilities in psychophysical tasks, a capacity which we did not find. Having all the discussed limitations of this cross-sectional study in mind, the reported findings at least suggest an association between expertise in mindfulness meditation and the subjective experience of the passage of time and of past duration. We can at least conclude that we captured meditators’ subjective experiences of time slowing down in everyday life.

## Conflict of Interest Statement

The Guest Associate Editor Sylvie Droit-Volet declares that, despite having collaborated with author Marc Wittmann, the review process was handled objectively and no conflict of interest exists. The authors declare that the research was conducted in the absence of any commercial or financial relationships that could be construed as a potential conflict of interest.
